# A Free N‐Heterocyclic Carbene and Its Metal Complex

**DOI:** 10.1002/anie.202524266

**Published:** 2026-03-26

**Authors:** Ankita Das, Tzu‐Chao Hung, Andreas Rank, Felix Giselbrecht, Jonas Schön, Mowpriya Das, Nikos Doltsinis, Saeed Amirjalayer, Jascha Repp, Frank Glorius

**Affiliations:** ^1^ Organisch‐Chemisches Institut University of Münster Münster Germany; ^2^ Institute of Experimental and Applied Physics University of Regensburg Regensburg Germany; ^3^ Institut für Festkörpertheorie and Center for Multiscale Theory and Computation University of Münster Münster Germany; ^4^ Interdisciplinary Center for Scientific Computing Heidelberg University Heidelberg Germany

**Keywords:** atomic force microscopy, N‐Heterocyclic carbenes, on‐surface characterization, scanning tunneling microscopy, ultrathin insulating films

## Abstract

Classical carbenes are highly reactive species that have traditionally been regarded as transient intermediates, making direct structural characterization a challenge. Among them, N‐heterocyclic carbenes (NHCs) stand out for their stability and broad applicability, yet atomic‐scale insights into their structure and electronic properties remain limited. Here, we report the on‐surface single‐molecule characterization of a free NHC deposited on ultrathin insulating NaCl layers on Au(111), enabling direct investigation of a reactive carbene in its free form. Using a combination of scanning tunneling microscopy (STM), atomic force microscopy (AFM), and density functional theory (DFT), we resolve its molecular structure and frontier orbital with sub‐molecular resolution. We further demonstrate the reactivity of the free NHC through its on‐surface complexation with a gold atom. These results demonstrate that real‐space techniques enable direct insights into the structure and reactivity of free NHCs, helping to connect molecular properties with their broader functional applications.

## Graphical abstract

Free N‐heterocyclic carbenes (NHCs) are directly visualized on ultrathin sodium‐chloride films on gold (NaCl/Au(111)) using scanning tunneling microscopy (STM), atomic force microscopy (AFM), and density functional theory (DFT). Submolecular imaging reveals their structure and frontier orbital. Furthermore, on‐surface reaction with a gold adatom forms an NHC–gold complex, characterized by the same techniques.

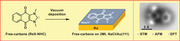

## 1 Introduction

Carbenes are defined as a class of neutral compounds containing a divalent carbon atom with six valence electrons [1]. Owing to their incomplete octet and coordinative unsaturation, carbenes have long been considered highly unstable and reactive species. After the pioneering isolation and characterization studies of carbenes by the groups of Bertrand and Arduengo, interest in carbenes surged dramatically [2, 3]. Stable (nucleophilic) carbenes since then have gained immense significance in various fields ranging from catalysis, material sciences, and biomedical applications [4, 5, 6, 7, 8].

**FIGURE 1:**
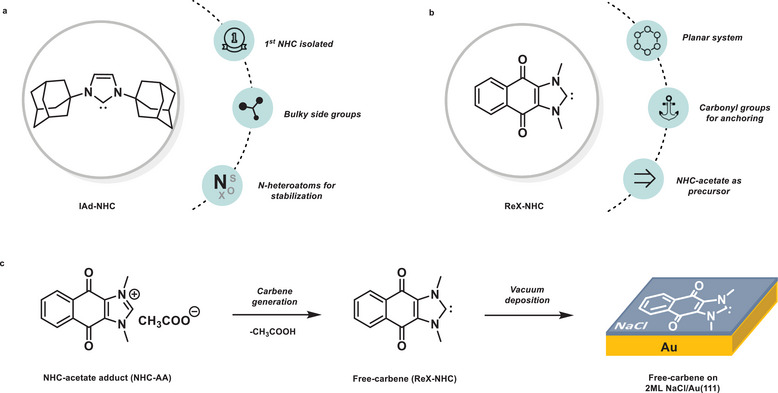
Overview of NHC systems relevant to this study. (a) First isolated NHC, IAd‐NHC. (b) Redox‐active NHC (ReX‐NHC) and its properties (system studied here). (c) The NHC‐acetate precursor releases the protecting group, converting into free carbene upon sublimation under ultra‐high vacuum (UHV) conditions, where it is deposited onto the cold NaCl/Au(111) surface (*T* < 8 K).

Synthesis and clear characterization of a free carbene had been a complex challenge, with the first documented attempt at isolating a carbene dating back as early as 1835 [9]. This conundrum was solved more than 150 years later in 1988 when Bertrand and coworkers first showed that the presence of heteroatoms could stabilize carbene species [3]. This was followed by the seminal work of Arduengo in 1991, where they reported synthesis and characterization of the first crystalline N‐heterocyclic carbene (NHC), the 1,3‐di(adamantyl)imidazol‐2‐ylidene (IAd‐NHC) (Figure 1a) [2]. They achieved this by incorporating the carbene carbon in a nitrogen‐containing heterocycle adorned with sterically demanding substituents, thereby fundamentally distinguishing NHCs—stable, isolable carbenes with well‐defined reactivity—from classical carbenes, which are typically highly reactive and observable only as transient intermediates [10]. Single‐crystal x‐ray diffraction has been one of the most used methods in identifying the detailed structural features of NHCs. Noteworthy was the valence angle of 102.2° at the carbene carbon, which was characteristic of singlet carbenes validated by theoretical models later [11]. Other techniques, like photoelectron spectroscopy [12] and nuclear magnetic resonance (NMR), along with other spectroscopic methods, were also applied in the structural determination of NHCs [13, 14, 15]. However, resolving the molecular structure using these methods depends on the unique response of the molecular ensemble to the applied stimuli, such as the electromagnetic radiation. Although real‐space imaging techniques at atomic resolution have been successfully used for various reactive species, their application to the structural characterization of free carbenes remains limited [16, 17, 18, 19]. A recent study by Sander, Sanchez‐Garcia, and Morgenstern demonstrated that real‐space imaging can be applied to visualize a metal surface*‐bound* electrophilic carbene (non‐NHC type). By combining scanning tunneling microscopy (STM) and theoretical analysis, they characterized the interaction of fluorenylidene with a silver surface [20]. While this work provided valuable insight into surface‐stabilized carbenes, the characterization of *free* carbenes remains largely unexplored. A major challenge in the structural characterization of free carbenes lies in their high intrinsic reactivity. While masking groups can stabilize them for analysis, this inevitably alters their electronic structure and reactivity. Therefore, gaining microscopic insight into the properties of free carbenes requires their immobilization in an inert environment.

Herein, we report the direct stabilization and real‐space imaging of a free NHC at the single‐molecule level. Using a combination of STM, atomic force microscopy (AFM), and density functional theory (DFT), we characterize the structure and electronic properties of a redox‐active free NHC. Individual molecules are stabilized at cryogenic temperatures by adsorption on an ultrathin insulating NaCl layer, enabling bond‐resolved visualization of both the molecular framework and its frontier orbital.

## 2 Results and Discussion

### 2.1 Rational Selection of the NHC and Its Precursor

The NHC studied here was meticulously chosen to align with the experimental environment of STM/AFM techniques applied to characterize it. In particular, we sought a molecule with an intrinsically planar π‐conjugated core and functional groups capable of providing additional stabilization upon adsorption on the 2ML NaCl/Au(111) surface. The redox‐active NHC (ReX‐NHC), featuring a quinone motif fused between an NHC ring and a benzene ring, fulfills these criteria (Figure 1b) [21]. Owing to its fully conjugated system of three fused rings, the molecular backbone adopts a planar geometry in isolation, while the quinone functionality promotes stable adsorption on the polar NaCl surface through weak, noncovalent interactions. Upon adsorption, minor out‐of‐plane distortions of peripheral substituents may occur, as discussed below, without compromising the overall planarity of the conjugated core. To enable the successful deposition of a free NHC under the required experimental conditions, a suitable precursor was required−one that remains stable during sublimation yet releases the carbene in situ before reaching the cold substrate surface. Carboxylate and bicarbonate adducts are widely used as carbene precursors, as they are known to generate free carbenes upon heating while producing benign byproducts such as CO_2_ and H_2_O, making them particularly attractive for controlled carbene generation [4, 12, 22, 23]. While bicarbonate and CO_2_ adducts are appealing from a chemical standpoint, in the present case, neither option was viable due to synthetic challenges and limited compatibility with the experimental conditions employed here.

Imidazolium acetate salts are widely used as organocatalysts and are known to generate free carbenes as a function of pressure and temperature, often referred to as “proto‐carbenes” [24, 25, 26, 27]. DFT calculations indicate reliable deprotonation in the gas phase, rendering it suitable for in situ sublimation (see Supplementary Information, Section 5) [28]. Therefore, we employed the corresponding imidazolium acetate as the carbene precursor. Specifically, we synthesized a redox‐active NHC‐acetate adduct (NHC‐AA) via anion‐exchange from the corresponding imidazolium salt using an anion‐exchange resin. This precursor releases free carbene upon sublimation under fast heating and ultra‐high vacuum conditions (Figure 1c; see Supplementary Information, Section 1.4).

### 2.2 On‐Surface Imaging and Characterization of ReX‐NHC

To characterize individual ReX‐NHC molecules, the carbene precursor (NHC‐AA) was thermally sublimed from a silicon wafer with native oxide and deposited onto a cold Au(111) single‐crystal surface covered with a few monolayers (ML) of NaCl at a sample temperature below 8 K. The NaCl layer acts as an inert electronic‐decoupling layer, allowing the molecule to be observed in its free state and enabling access to its intrinsic electronic properties [29]. Sublimation of the precursor yields free ReX‐NHC on the surface, along with acetic acid as a byproduct. Overview STM images (Supplementary Figure 1) reveal two distinct types of features, indicating the presence of two molecular species, ReX‐NHC and the released protecting group. One of these features exhibits a dumbbell shape, which we attribute to the acetic acid byproduct formed during the deprotection process (See Supplementary Figure 2 for characterization of acetic acid).

**FIGURE 2:**
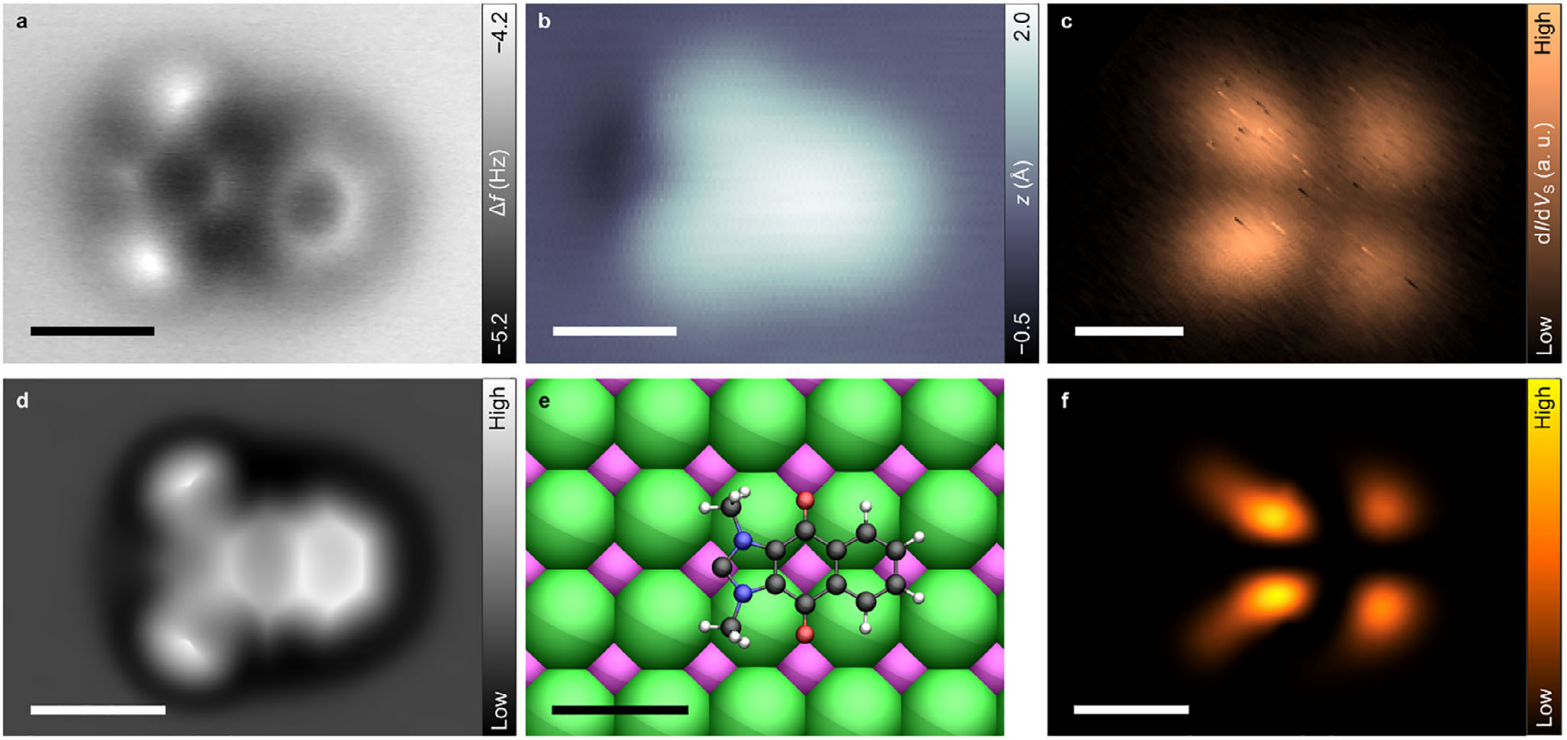
Characterization of single‐isolated ReX‐NHC on 2 ML NaCl/Au(111). (a) Bond‐resolved constant‐height Δ*f* image of the ReX‐NHC (*V*_S_ = 0 V, Δ*z* = 0.46 Å). The NHC group can be discriminated from the benzene ring at the opposing end of the molecule due to the pentagonal shape of the former, as well as the two methyl groups. The carbene site exhibits a sharp and point‐like feature, which differs from all other carbon atoms. (b) Constant‐current STM image of ReX‐NHC (*I* = 0.5 pA, *V*_S_ = 0.2 V). A dark depression can be seen at the carbene position, suggesting a localized electron‐rich region (see main text). (c) Constant‐height d*I*/d*V*_S_ map of the negative ion resonance (*V*_S_ = 1.2 V). (d) Simulated AFM image of the ReX‐NHC based on the probe particle model^30^. (e) Calculated lowest‐energy adsorption site of the ReX‐NHC on NaCl (color code: Na^+^ magenta, Cl^−^ green). (f) Calculated constant‐height electron density map of the LUMO of ReX‐NHC adsorbed on NaCl (See Supplementary Information, Section 4 for computational details). All scale bars refer to 5 Å.

Figure 2a shows a bond‐resolved constant‐height AFM image, acquired with a CO‐functionalized tip [30], identified in the following as a single‐isolated ReX‐NHC. The cyclic scaffold of this species appears to be adsorbed parallel to the surface plane, while revealing a saddle‐like distortion in the AFM image. As such images predominantly reflect the molecular geometry, this suggests that the carbonyl groups anchor to the NaCl surface as intended, pulling the central carbon ring toward the surface [31]. Such features were observed and characterized more than ten times. Since bond‐resolving AFM is very sensitive to vertical displacements, the contrast indicates a shallow puckering of the molecular backbone rather than a strong deformation. NaCl is a wide‐band‐gap insulator and has no electronic states available in a large energy range around the chemical potential of the system; thus, only noncovalent interactions occur between ReX‐NHC and the underlying NaCl. The overall adsorption of ReX‐NHC on NaCl is expected to be governed by van der Waals interactions, with additional spatially confined electrostatic interactions between the polar carbonyl groups and surface ions, stabilizing the adsorption site and orientation. The latter is consistent with the experimental adsorption‐site determination (Supplementary Figure 3). The above is corroborated by DFT calculations, which show a clear energetic preference for configurations in which the carbene carbon is positioned above a Na^+^ ion rather than a Cl^−^ ion (Supplementary Figure 9). The lowest‐energy configuration, consistent with the experimentally determined adsorption site, is shown in Figure 2e. Importantly, no covalent bonding is observed between ReX‐NHC and the NaCl substrate in the charge redistribution (Supplementary Figure 10), consistent with the aforementioned reasons for using NaCl as an electronically decoupling layer.

The NHC group can be readily discriminated from the benzene ring at the opposing end of the molecule due to the pentagonal shape of the former, as well as the two methyl groups. These methyl groups, which are not part of the conjugated backbone, adopt slightly out‐of‐plane orientations upon adsorption, leading to enhanced repulsive contrast in the AFM images. From this structural characterization, shown in Figure 2a, the carbene site can be readily identified (for a discussion of protonation of the carbene site, see Supporting Information, Section 5). Interestingly, the carbene site exhibits a sharp and point‐like feature, which differs from all other carbon atoms, for example, in the benzene ring of the backbone. This feature could be related to the carbene's unusual chemical nature. Based on the calculated electrostatic potential of the DFT‐optimized structure, AFM images were simulated using the probe particle model (Figure 2d) [32]. The simulated images show qualitative agreement with the measured data. The remaining discrepancies likely arise from the limitations of DFT‐level calculations in fully accounting for van der Waals interactions, which may influence the precise geometry—particularly between oxygen and sodium—and contribute to the molecule's nonplanarity (see Supplementary Figure 9 and Supplementary Information, Section 6 for detail).

In low‐voltage STM constant‐current images (Figure 2b), the carbene position is imaged as a depression. Similar features have been reported for molecules adsorbed on NaCl films, where depressions in low‐voltage STM images are associated with electron‐rich regions [33, 34] and under‐coordinated carbon sites [35, 36] of planar organic molecules. Both properties are consistent with those of a free carbene [1]. In addition, our DFT calculations did not show any evidence for a covalent (*V*_S_ = 1.2 V) bond formation between ReX–NHC and the NaCl surface, indicating a predominantly electrostatic interaction of the NHC with the substrate. This noncovalent adsorption stabilizes the molecule without significantly perturbing its geometry or electronic structure (Supplementary Figure 10).

To gain deeper insight into the electronic structure of the single, isolated ReX‐NHC, we conducted differential‐conductance mapping to investigate the densities of frontier molecular orbitals [29]. Figure 2c shows a constant‐height differential conductance image acquired at a sample bias voltage of *V*_S_ = 1.2 V corresponding to the negative ion resonance (NIR) [29]. The pixel‐scale noise‐like fluctuations in the experimental images in Figure 2c, we associate with minute variations of the molecular geometry induced by the tunneling current [37, 38, 39, 40], for example, slight rotations of the methyl groups. To scrutinize our assignment and to mimic the measurements at constant‐height, the electron density associated with the lowest unoccupied molecular orbital (LUMO) was integrated along the coordinate axis perpendicular to the surface over a distance of 2.4 Å, starting approximately 3 Å above the molecule, and then projected onto the surface plane. The resulting density map (Figure 2f) exhibits comparable contrast to the experimental measurements, supporting the assignment to the LUMO. The positive ion resonance (PIR), corresponding to tunneling out of the highest occupied molecular orbital (HOMO), was not observed in our measurement down to voltages of *V*_S_ = −4 V. The absence of a clear PIR signature could be due to a high ionization energy resulting from the strong localization of the HOMO and its associated large Coulomb charging energy. The theoretical analysis of the molecular orbitals of ReX‐NHC on the NaCl surface shows that its electronic character (spatial distribution) remains unchanged. This suggests that the interaction between the ReX‐NHC molecule and the surface does not alter the fundamental nature of the LUMO—in line with the nonchemical nature of this interaction, such that the molecule is only physisorbed.

### 2.3 On‐Surface Reaction between the ReX‐NHC and Gold Adatom

**FIGURE 3:**
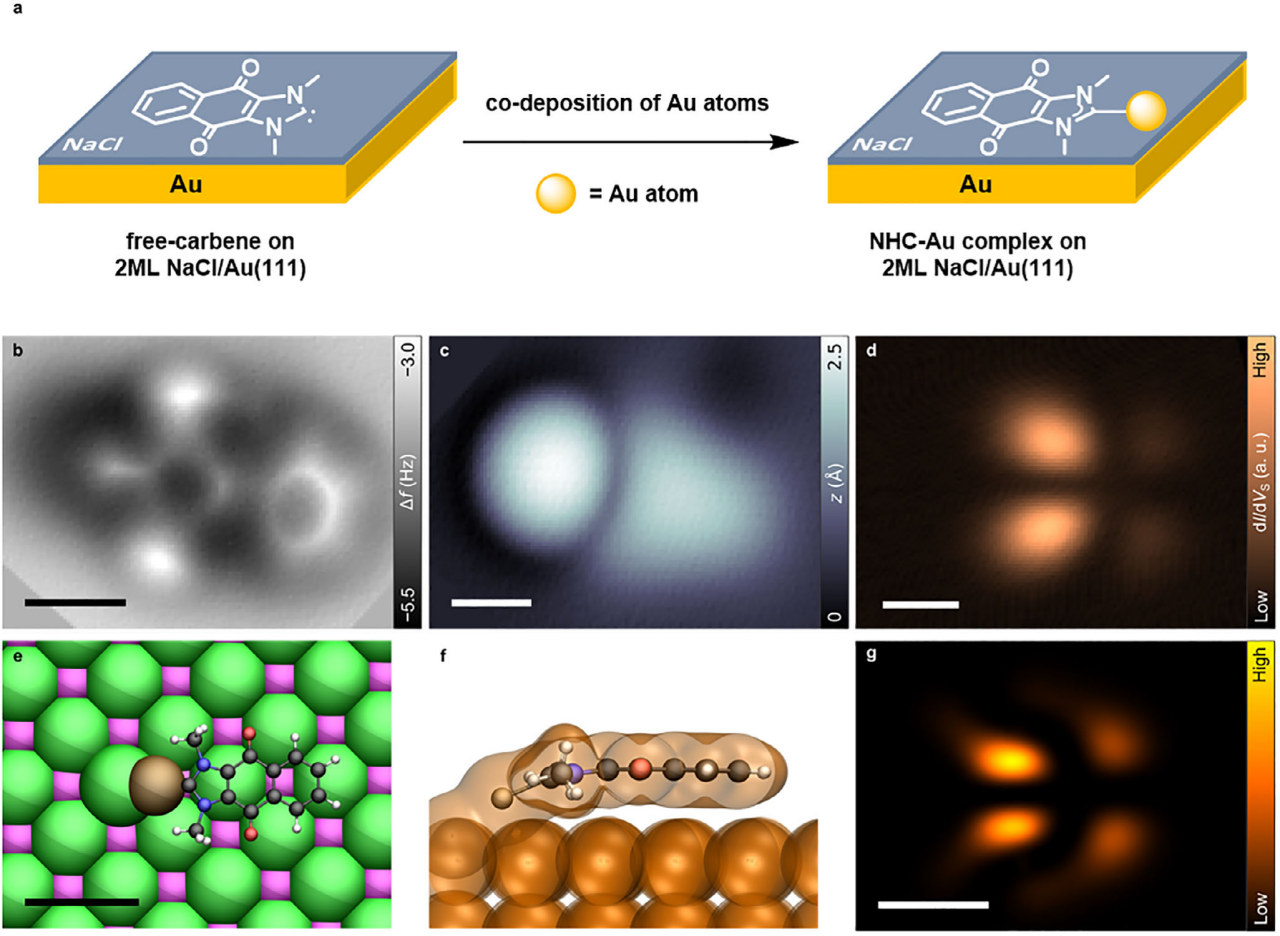
Characterization of Au‐ReX‐NHC on 2 ML NaCl/Au(111). (a) Reaction scheme. (b) Bond‐resolved constant‐height Δ*f* image of the Au‐ReX‐NHC (*V*_S_ = 0 V, Δ*z* = −1.15 Å) shows a cyclic scaffold as shown in Figure 2a for isolated ReX‐NHC, but with a bond connecting to the Au atom from the carbene position of ReX‐NHC. (c) Constant‐current STM image of Au‐ReX‐NHC (*I* = 0.5 pA, *V*_S_ = 0.2 V) in which the Au atom appears as a protrusion attaching to the carbene site of the molecule. (d) Constant‐height d*I*/d*V*_S_ map of the negative ion resonance (*V*_S_ = 1.0 V). (e) Calculated lowest‐energy adsorption site of Au‐ReX‐NHC on NaCl (color code: Na^+^ magenta, Cl^−^ green). (f) Side view of optimized geometry Au‐ReX‐NHC on NaCl, indicating Au atom binding to both a Cl atom and to ReX–NHC. As a result, the Au‐carbene bond points toward the surface with respect to the backbone of ReX‐NHC. The corresponding calculated electron density (isovalue: 0.01 e/Å^3^) is shown. (g) Calculated constant‐height electron density map of the SOMO of Au‐ReX‐NHC adsorbed on NaCl (See Supplementary Information, Section 4 for computational details). All scale bars refer to 5 Å.

To gain further insights into the reactivity of the free ReX‐NHC, we investigated its on‐surface reaction [41] on the ultrathin NaCl/Au(111) surface (Figure 3a). Due to the unique structural and electronic features, NHCs can act as σ‐donors. This characteristic confers a strong affinity for binding to metal centers, which in turn underlies the widespread use of NHC‐ligated metal complexes in catalytic cycles and the use of NHCs as surface modifiers [42]. Notably, the NHC‐Au bond has been widely studied in various contexts, including NHC‐Au complexes [43, 44], NHC‐stabilized Au‐clusters [45, 46], nanoparticles [47], and even self‐assembled monolayers (SAMs) of NHC on Au‐surfaces [22, 48, 49], the real‐space observation of the covalent bond formation between a free NHC and a gold atom has remained elusive.

To this end, we codeposited Au adatoms onto the NaCl(2ML)/Au(111) surface, kept at low temperature (*T* < 8 K). The Au adatoms appeared as circular protrusions with an apparent height of about 2.4 Å (Supplementary Figure 4) [50]. After deposition of Au, in addition to isolated Au and ReX‐NHC adsorbates, another species was observed (red‐dashed oval in Supplementary Figure 4). A low‐voltage STM constant‐current image of such a feature is shown in Figure 3c, identified in the following as an Au‐ReX‐NHC, in which the Au atom appears as a protrusion attaching to the carbene site of the molecule. Such features were observed and characterized seven times. A bond‐resolved constant‐height AFM image (Figure 3b) of this species, acquired with a CO‐functionalized tip, shows a cyclic scaffold as shown in Figure 2a for isolated ReX‐NHC, but with an extra feature at the carbene site, which we assign as the covalent bond connecting to the Au atom from the carbene position of ReX‐NHC. The circular, shallow, and more negative AFM signal observed at the terminus of the covalent bond can be attributed to van der Waals attraction between the Au atom and the CO‐functionalized tip apex. In addition, it could be formed by applying voltage pulses if—by chance—an Au adatom was adsorbed already in close vicinity to the carbene site of ReX‐NHC. Further evidence for a covalent bond between the Au atom and ReX‐NHC in the structures identified as Au‐ReX‐NHC is provided by STM manipulation experiments: Current and voltage pulses applied to Au‐ReX‐NHC could lead to rotation of the ReX‐NHC around the Au atom and displacement of the entire structure but neither Au charging nor bond breaking was observed (Supplementary Figures 5 and 6, respectively) [50, 51]. The adsorption geometry of the Au‐ReX‐NHC was determined experimentally (see Supplementary Figure 7) and is consistent with DFT calculations shown in Figure 3e. The optimized geometries show that the Au atom, sitting on a Cl‐Na bridge site, binds to the ReX‐NHC. The Au‐Cl interaction results in the Au‐carbene bond pointing toward the surface with respect to the backbone of ReX‐NHC (Figure 3f).

Figure 3d shows a constant‐height differential‐conductance image acquired on Au‐ReX‐NHC at a sample bias voltage of *V*_S_ = 1.0 V corresponding to the NIR [29]. The nodal plane structure is in agreement with the integrated electron density of the calculated singly‐occupied molecular orbitals (SOMO) for the adsorbed Au‐ReX‐NHC (Figure 3g). A PIR signature was not observed down to a voltage of *V*_S_ = −3 V; probably for the same reason as discussed for ReX‐NHC, see above.

The observed coordination of the NHC to the Au atom is consistent with the well‐established strong σ‐donor interaction between free carbenes and gold [41], providing additional evidence that the characterized species is indeed a free carbene.

## 3 Conclusion

To conclude, we successfully characterized a single, free NHC on a bilayer NaCl/Au(111) surface by means of scanning probe microscopy and demonstrated its on‐surface reaction with a gold atom, leading to the formation of a carbene–gold complex. Our study builds on the powerful combination of real‐space imaging and theoretical analysis to investigate the structure and reactivity of reactive intermediates at the single‐molecule level [16, 18, 19]. By studying the carbene immobilized on an ultrathin insulating film under ultrahigh vacuum, we access a limiting case in which the NHC is free from solvent and crystal packing effects. In contrast to solution‐phase studies, where “free” NHCs are inherently influenced by continuous interactions with the surrounding medium, this approach enables characterization of the intrinsic geometric and electronic structure of an isolated carbene and allows its stepwise coordination to a metal atom to be followed within the same experimental framework. It extends this atomistic surface‐based approach to the rich and contemporary chemistry of free NHCs and their interactions with metal atoms as a unique complement to traditional solution‐phase techniques, providing new perspectives. Given the wealth of literature on the applicability of NHCs, from catalysis to surface chemistry and molecular electronics, atomic‐scale insights into their intrinsic properties and reactivity can inform the design and optimization of NHC‐based systems, for example, in applying NHC‐stabilized metal complexes in catalysis in both homogeneous and heterogeneous systems.

## Conflicts of Interest

The authors declare no conflicts of interest.

## Supporting information



Detailed descriptions of the synthesis and characterization, experimental procedures, and additional data supporting the findings of this study are included. Computational details are also included. All other data are available from the corresponding author upon reasonable request.
**Supporting File**: anie71956‐sup‐0001‐SuppMat.pdf.

## Data Availability

Data available in the article's supplementary material. The data that support the findings of this study are available in the supplementary material of this article.
