# Supplementary material for: A Free N‐Heterocyclic Carbene and Its Metal Complex
Source: Angew Chem Int Ed Engl. 2026 Mar 26;65(19):e24266. doi: 10.1002/anie.202524266 (PMC13134596; doi:10.1002/anie.202524266)
Supplement: Supplementary file 1 — Detailed descriptions of the synthesis and characterization, experimental procedures, and additional data supporting the findings of this study are included. Computational details are also included. All other data are available from the corresponding author upon reasonable request. Supporting File: anie71956‐sup‐0001‐SuppMat.pdf. [file ANIE-65-e24266-s001.pdf]

Supplementary Materials

A Free N-Heterocyclic Carbene and its Metal Complex

Ankita Das[1,‡], Tzu-Chao Hung[2,‡], Andreas Rank[2], Felix Giselbrecht[2], Jonas Schön[2], Mowpriya Das[1], Nikos Doltsinis[4], Saeed Amirjalayer[3]*, Jascha Repp[2]*, Frank Glorius[1]*

[1]Organisch-Chemisches Institut, University of Münster; Corrensstraße 36, 48149 Münster, Germany;

[2]Institute of Experimental and Applied Physics, University of Regensburg, Universitätsstr. 31, 93040 Regensburg, Germany;

[3]Interdisciplinary Center for Scientific Computing, Heidelberg University, Im Neuenheimer Feld 205A, 69120 Heidelberg;

[4]Institut für Festkörpertheorie and Center for Multiscale Theory and Computation, University of Münster; Wilhelm-Klemm-Straße 10, 48149 Münster, Germany.

[‡]These authors contributed equally to this work.

*Email: glorius@uni-muenster.de (F.G.), jascha.repp@ur.de (J.R.) and saeed.amirjalayer@iwr.uni-heidelberg.de (S.A.)

Contents

1. Synthesis....S1

1.1 General Information....S1

1.2 Synthesis of Redox-Active NHC-Acetate....S2

1.3 NMR Spectra....S5

1.4 Direct-Inlet Spectra of ReX(H)-CH_3CO_2 Precursor....S7

2. Sample Preparation....S8

3. STM/AFM Measurements....S8

3.1 Additional Scanning Probe Microscopy Figures....S9

4. Computational Details....S17

4.1 Additional Computational Data....S18

5. Discussion of Protonation....S21

6. Comparison of Simulated and Experimental AFM Images....S22

7. References....S24

1. Synthesis

1.1 General Information

All reactions were carried out in oven-dried glassware with oven-dried Teflon-coated magnetic stir bars. Dry solvents were either taken from a solvent purification system (HPLC grade, dried over activated alumina columns) or purchased from Acros Organics, Sigma-Aldrich or Carl Roth (stored over activated molecular sieves). All reagents were obtained from ABCR, Acros Organics, Alfa Aesar, Carbolution Chemicals, Carl Roth, Chempur Combi-Blocks, Fisher Scientific, Fluorochem, Merck, Sigma-Aldrich, TCI Europe or VWR and utilized as received.

1H- and 13C-NMR spectra were recorded on a Bruker AV 400 at room temperature. Chemical shifts (δ) were given in ppm. The residual solvent signals were used as references and the chemical shifts converted to the TMS scale (MeOD: δH = 4.87 ppm, δC = 49.00 ppm; CD_2Cl_2: δH = 5.32 ppm, δC = 54.00 ppm; D_2O: δH = 4.79 ppm,). All the NMRs were processed using Mestrenova 14 applying standard phase and baseline corrections. Coupling constants (J) are quoted in Hz.

High resolution mass spectra (HRMS) were recorded on a Thermo Scientific Exploris 120 Electrospray Orbitrap in electrospray ionizsation mode (ESI). ESI spectra show relative abundance after normalization against maximum signal intensity level (NL) in dependence of m/z.

Direct inlet electron impact mass spectra with temperature profile (Direct inlet EIMS) were performed on a Thermo Scientific TSQ 7000 a pressure of 1.99 to 2.66 × 10^{-7} mbar. The ionization mode was electron ionization with an electron acceleration of 70 eV. The cathode was cooled by an internal water-based cooling system to around 32 °C at the beginning of the experiment and heated up consequently to around 250 °C. The solid material was placed in a clean crucible, transferred into the high vacuum of the spectrometer and heated from room temperature to 400 °C to follow the evaporation of the “free” NHC.

1.2 Synthesis of Redox-Active NHC-Acetate

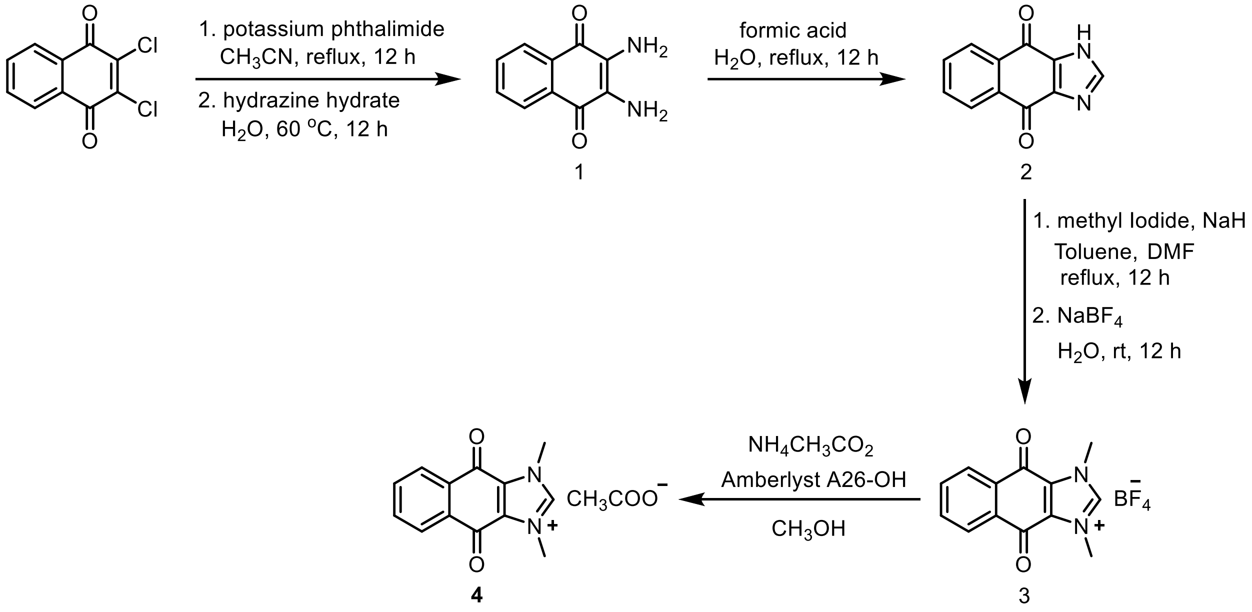

Scheme 1. Synthesis of NHC-acetate adduct [ReX(H)CH_3CO_2].

2,3-Diaminonaphthalene-1,4-dione (1)

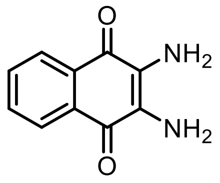

2,3-dichloro-1,4-naphtoquinone (2.26 g, 10.0 mmol, 1.0 equiv) was dissolved in CH_3CN (46 mL) and potassium phtalimide (3.88 g, 21.0 mmol, 2.1 equiv) was added to the mixture. The solution was refluxed under argon for 16 h. After cooling to room temperature, yellow solid was collected by filtration and further dried under vacuum. The dried yellow solid was suspended in distilled water (50 mL) and 2.70 mL of hydrazine hydrate was added. After reaction was stirred overnight at 60 °C, a dark blue-violet powder was formed. This solid was collected after filtration and washing with distilled water. After drying the desired compound is obtained (1.62 g, 86.17 mmol, 86 % yield).

^{1}H NMR (400 MHz, DMSO) δ (ppm): δ 7.8 – 7.7 (m, 2H), 7.6 – 7.5 (m, 2H), 5.4 (s, 4H).

^{13}C NMR (101 MHz, DMSO) δ (ppm): δ 178.4, 132.6, 131.1, 127.6, 124.6.

HRMS (ESI+): m/z calculated for $C_{10}H_8N_2O_2Na^+$ $[M+Na]^+$: 211.0477; found: 211.0478

1*H*-naphtho[2,3-d]imidazole-4,9-dione (2)

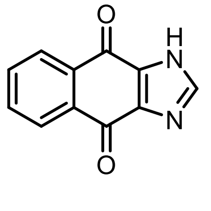

To synthesize the titled compound, diamine **1** (1.6 g, 8.5 mmol, 1.0 equiv.) was suspended in 15 mLformic acid and stirred at 110 ^{o}C for overnight. After completion of the reaction, distilled water (20 mL) was added resulting in a yellow precipitate. To neutralize the excess formic

acid, ammonium acetate was added until pH 7 is reached. The resulting brown solution was filtered and the precipitate was washed with distilled water. The desired product was obtained as a muddy yellow solid (1.53 g, 0.89 mmol, 89 %).

^{1}H NMR (400 MHz, DMSO) δ (ppm): δ 8.2 (s, 1H), 8.1 (dt, J = 7.5, 3.8 Hz, 2H), 7.8 (dt, J = 5.7, 3.6 Hz, 2H).

^{13}C NMR (101 MHz, DMSO) δ (ppm): δ 177.3, 144.3, 139.3, 133.7, 133.0, 126.2.

HRMS (ESI+): m/z calculated for $C_{11}H_6N_2O_2Na^+$ $[M+Na]^+$: 221.0319; found: 221.0321

1,3-Dimethyl-4,9-dioxo-4,9-dihydro-1H-naphtho[2,3-d]imidazole-3-ium tetrafluroborate (3)

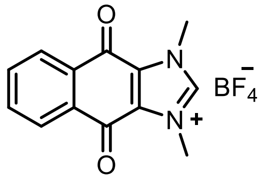

To synthesize the titled compound **3,** the imidazole **2** (735 mg, 3.7 mmol, 1.0 equiv.) was taken in Schleck flask then toluene (28 mL) and sodium hydride (60 % in mineral oil) (252.4 mg, 6.31 mmol, 1.7 equiv.) was added. After stirring for 4 h at room temperature, methyl iodide (0.92 mL, 14.8 mmol, 4.0 equiv.) in 28 mL DMF was slowly added to the reaction mixture. Then it was stirred overnight at 110 ^{o}C. After cooling the reaction mixture to room temperature, diethyl ether was added to result an off-white precipitate. The precipitate was filtered and vacuum dried before being dissolved in 200 mL distilled water and then $NaBF_4$ (446 mg, 4 mmol, 1.1 equiv.) was added. The reaction was stirred for 4-5 h until red precipitate was formed. The precipitate was filtered and washed with water. The final compound was obtained as red solid after vacuum drying (198 mg, 0.63 mmol, 17 %).

^{1}H NMR (400 MHz, DMSO) δ (ppm): δ 9.6 (s, 1H), 8.3-8.2 (m, 2H), 8.1 – 8.0 (m, 2H), 4.2 (s, 6H).

^{13}C NMR (101 MHz, DMSO) δ (ppm): δ 174.9, 143.8, 135.3, 131.8, 130.8, 126.9, 36.1.

HRMS (ESI+): m/z calculated for $C_{13}H_{11}N_2O_2^+$ $[M]^+$: 227.0814; found: 227.0815

1,3-Dimethyl-4,9-dioxo-4,9-dihydro-1H-naphtho[2,3-d]imidazol-3-ium acetate (4)

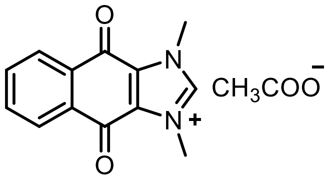

The titular compound was synthesized using a modified literature procedure.[1] A fritted column for silica chromatography was filled with ion exchange resin (Amberlyst A26-OH, 20.0 g). $NH_4CH_3CO_2$ (6.00 g) was

dissolved in water (300 mL) and the column was flushed with aq. $NH_4CH_3CO_2$ solution until the pH of the eluted solvent was about 9. Afterwards, the column was flushed with MeOH (100 mL, 2 times), while after the last cycle, the column was gently rotated to remove air bubbles from the resin material and ensure appropriate packing. After the settlement of all resin material, the solvent was eluted to the level of resin in the column. The corresponding imidazolium tetrafluoroborate salt (100 mg, 0.318 mmol, 1.0 eq.) was dissolved in methanol (5 mL) and added onto the resin. The solvent was eluted to let the solution penetrate the resin material, then the column was refilled with MeOH (100 mL). The solvent was collected in reaction tube fractions and checked for the elution of the imidazolium salt via TLC plate staining under UV light (254 nm) exposure. The positive fractions were combined and the solvent was removed by rotary evaporation (temperature should not exceed 30 °C). Drying of the remaining residue in an oil pump vacuum (< 1 mbar) lead to crystallization of an orange solid (40.0 mg, 0.140 mmol, 44%). For regeneration, the column was washed with aq. KOH solution (4.00 g in 200 mL H_2O) until the pH was 14.

^{1}H NMR (400 MHz, D_2O) δ (ppm): δ 8.2– 8.16 (m, 2H), 7.9– 7.8 (m, 2H), 4.25 (s, 6H), 1.91 (s, 3H).

^{13}C NMR (101 MHz, D_2O) δ (ppm): δ 180.1, 176.0, 135.5, 131.7, 131.3, 127.3, 36.0, 22.4.

HRMS (ESI+): m/z calculated for $C_{13}H_{11}N_2O_2^+$ $[M]^+$: 227.0814; found: 227.0815.

Direct inlet EIMS: m/z calculated for $C_{13}H_{10}N_2O_2^{\cdot+}$= $[M]^{\cdot+}$: 226.1, at 70 (±20) °C found: $[M]^{\cdot+}$: 226.1

1.3 NMR Spectra

1.3.1. 1,3-dimethyl-4,9-dioxo-4,9-dihydro-1H-naphtho[2,3-d]imidazole-3-ium tetrafluroborate

^{1}H spectrum (400 MHz, DMSO-d6)

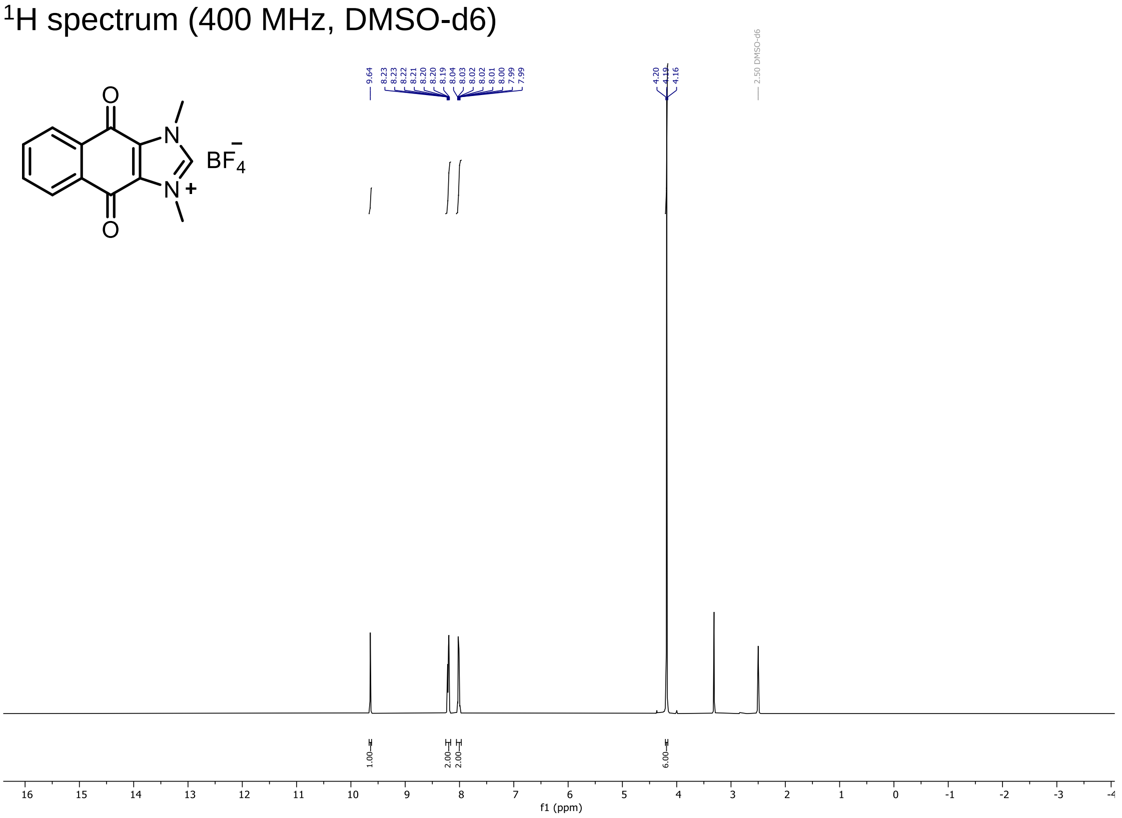

^{13}C spectrum (100 MHz, DMSO-d6)

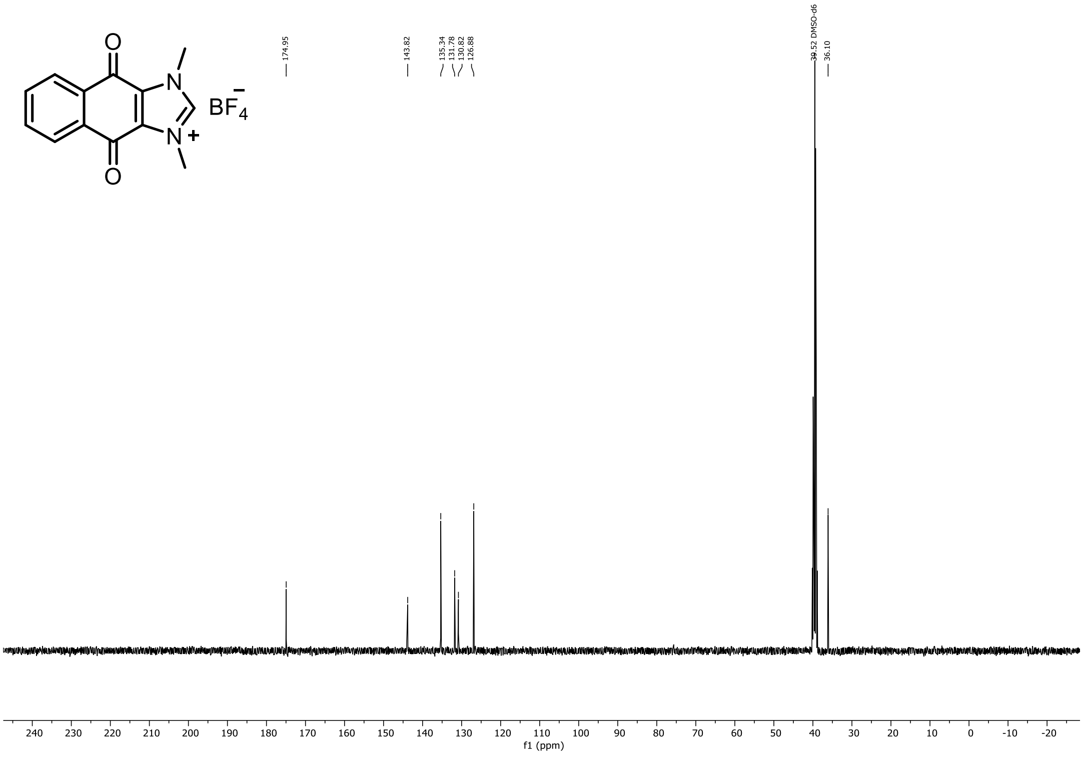

1.3.2. 1,3-dimethyl-4,9-dioxo-4,9-dihydro-1H-naphtho[2,3-d]imidazol-3-ium acetate

^{1}H spectrum (400 MHz, D_2O-d2)

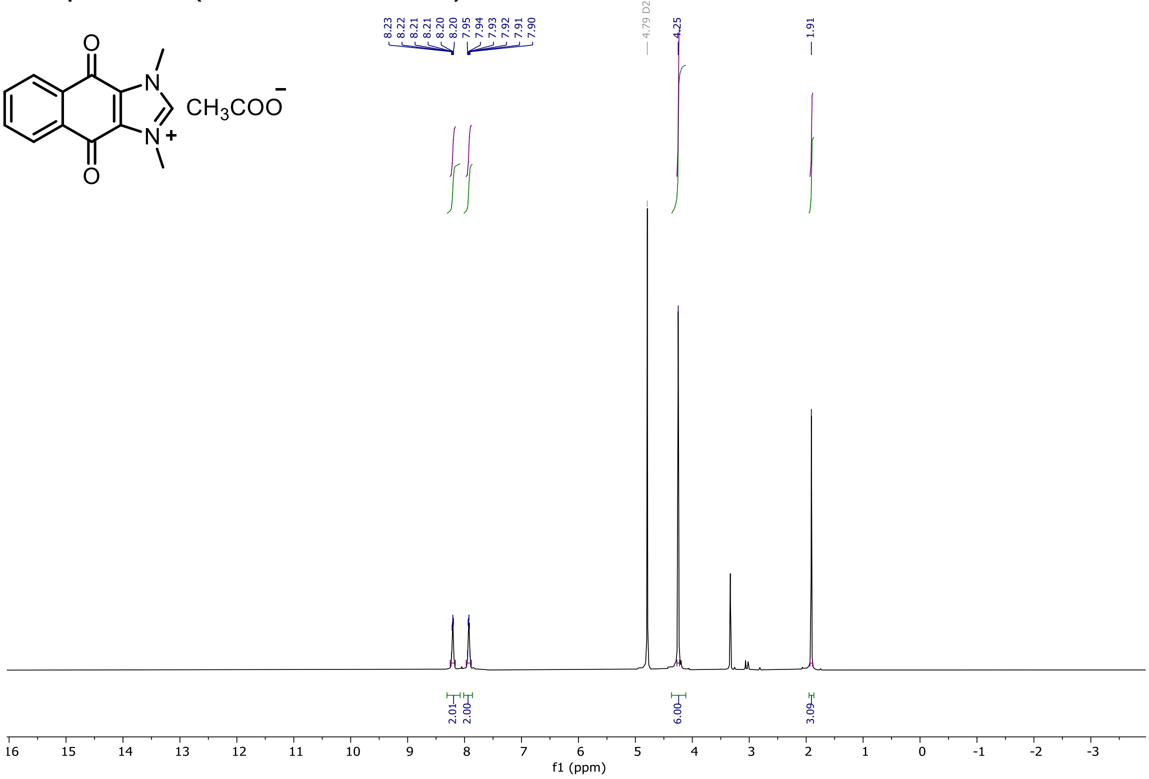

^{13}C spectrum (100 MHz, D_2O-d2)

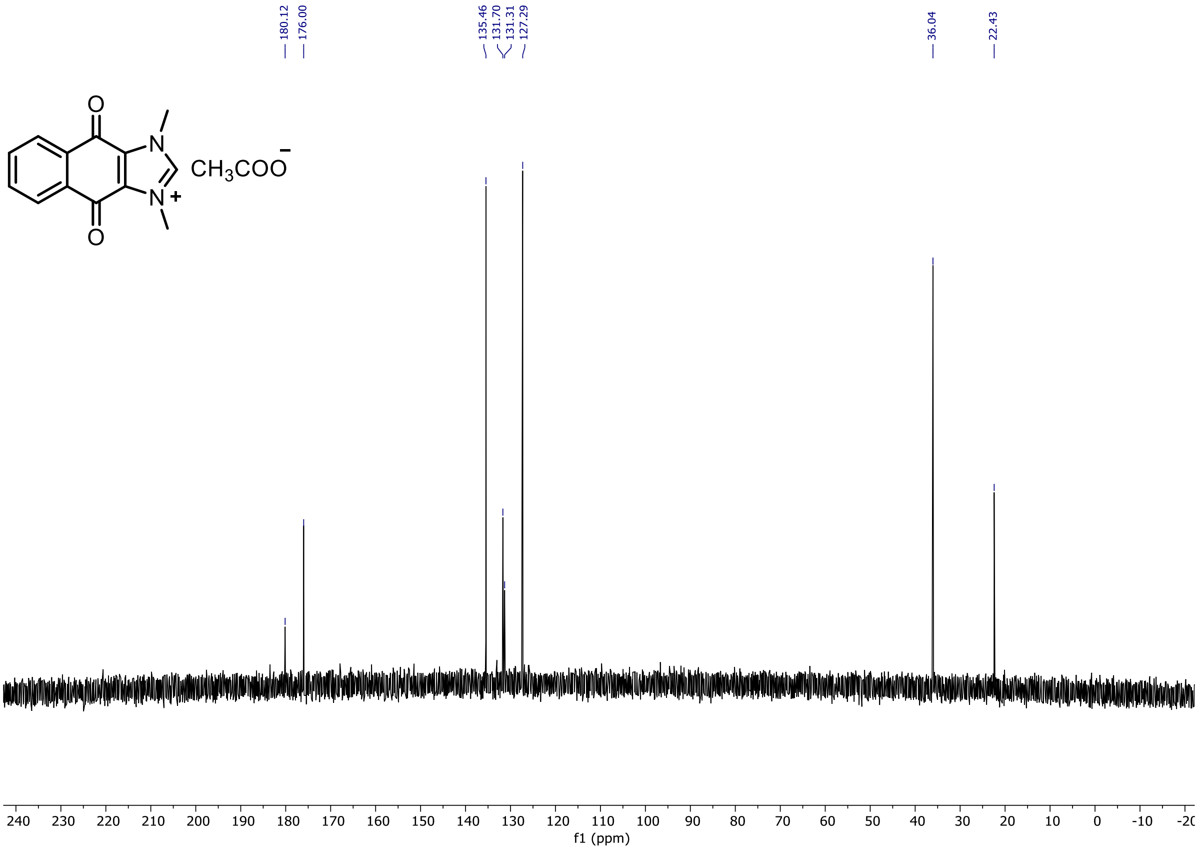

1.4 Direct-Inlet Spectra of ReX(H)-CH_3CO_2 Precursor

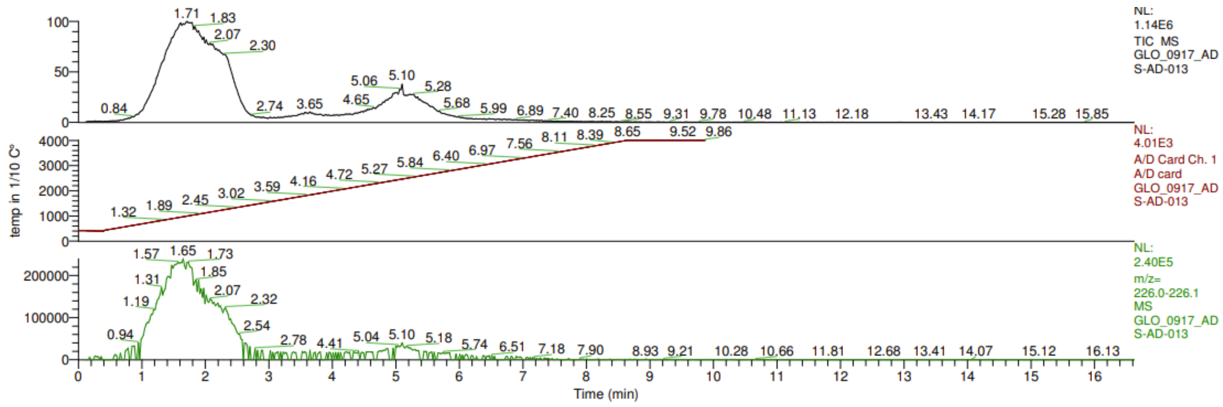

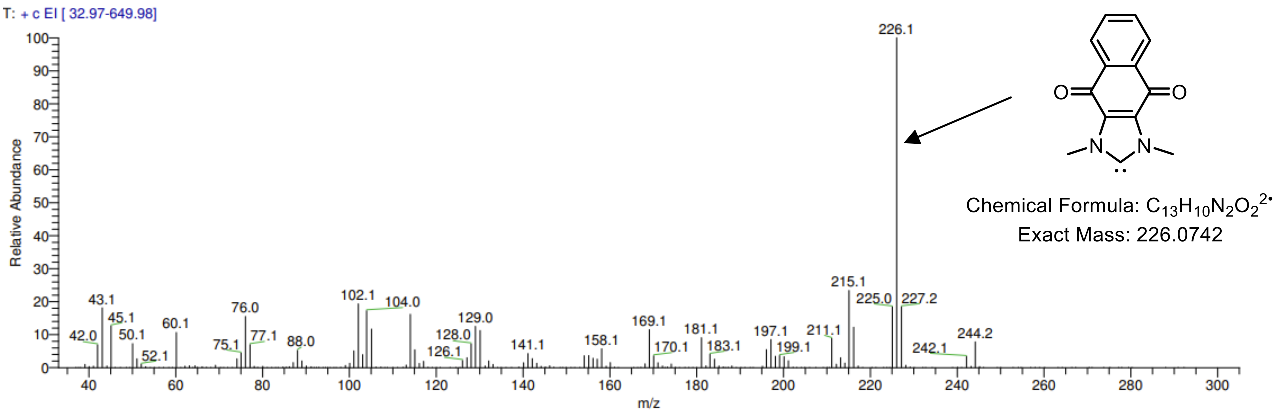

2. Sample Preparation

Au(111) single-crystal surface was cleaned by several sputtering and annealing cycles. NaCl was deposited onto the cleaned Au(111) surface, kept at room temperature, to form islands of 2-3 atomic layers in thickness which is referred as 2-3 monolayers (ML) NaCl in the main text.

To characterize individual ReX-NHC molecules, the carbene-precursor (NHC-AA) was thermally sublimed from a silicon-wafer with native oxide and deposited onto the cold Au(111) sample (T < 8 K), covered with few ML NaCl islands, in the STM head.

The Au adatoms were thermally sublimed from a home-built gold evaporator source (~1mm gold sphere in a W filament) and deposited onto the cold sample with ReX-NHC on top (T < 10 K). The overview STM image after the deposition is shown in Supplementary Figure 4. The Au adatoms adsorbed on 2 ML NaCl appeared as circular protrusions with an apparent height about 2.4 Å.

3. STM/AFM Measurements

All the STM/AFM measurements were conducted in a home-built STM/AFM setup, operated at a temperature of 7 K and a base pressure below $1 \cdot 10^{-10}$ mbar. qPlus sensors[2] were used in the measurements. Figure 2a was measured with a sensor with resonance frequency, f_0 = 27974 Hz, quality factor, Q ~ 25,000, and oscillating amplitude, A = 2 Å. Figure 3b was measured with a sensor with f_0 = 28007 Hz, Q ~ 130,000, and A = 1 Å. For the constant-height AFM images, the feedback loop was opened at the set-point of I_{SP} = 0.5 pA, V_{SP} = 0.2 V above 2 ML NaCl followed with a vertical tip displacement of Δz, as indicated. Positive values of Δz refer to the tip being closer to the surface. The constant-height differential conductance (dI/dV_S) maps were acquired with lock-in technique with modulation frequency (f_{mod}), and modulation voltage ($V_{mod,pp}$). Figure 2c was acquired with parameters of $V_{mod,pp}$ = 10 mV, f_{mod} = 289 Hz, I_{SP} = 10 pA, V_{SP} = 1.25 V, Δz = 0 Å. Figure 3d was acquired with parameters of $V_{mod,pp}$ = 20 mV, f_{mod} = 378 Hz, I_{SP} = 2 pA, V_{SP} = 1.20 V, Δz = −2 Å. CO-functionalized tips[3] were used in all the bond-resolved AFM images and Figure 2b, otherwise metal tips were used

3.1 Additional Scanning Probe Microscopy Figures

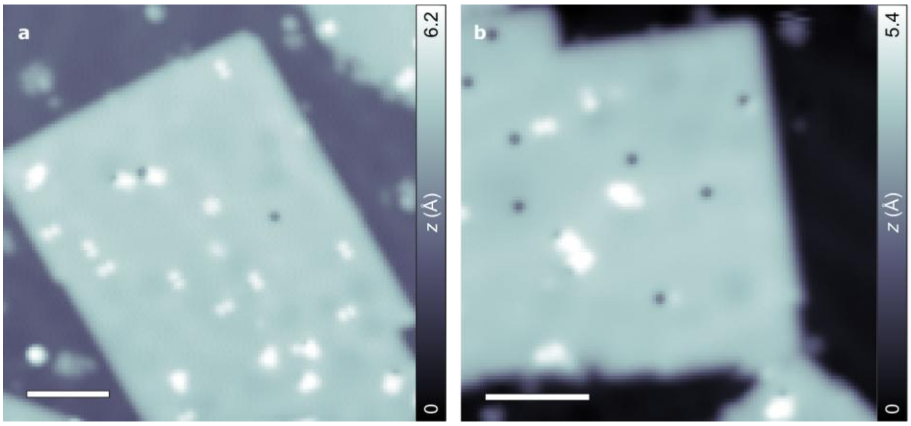

Supplementary Figure 1. Sample overview. **a**, Constant-current STM image (I = 0.5 pA, V_S = 0.2 V) shows numerous features adsorbed on 2 ML NaCl/Au(111). CO molecules appeared as circular depressions, and the dumbbell-shape features are attributed to the acetate protecting group. The ReX-NHC appeared as an elongated heart shape with a depression at one end (Figure 2b for detail). **b**, Constant-current STM image (I = 1.0 pA, V_S = 0.2 V) acquired on a different surface area. All scale bars refer to 5 nm.

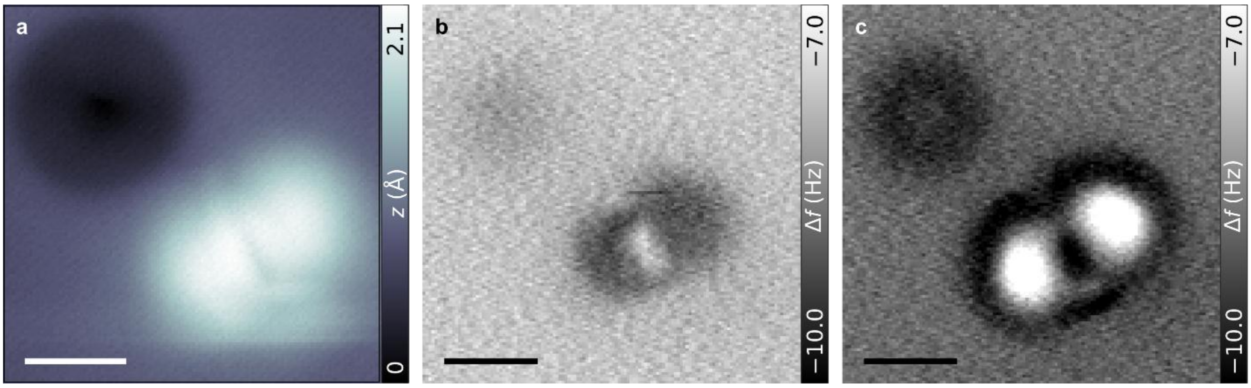

Supplementary Figure 2. Characterization of acetic acid on 2 ML NaCl/Au(111). **a**, Constant-current STM image of the secondary, but predominant adsorbate (byproduct formed during the deposition process) showing a dumbbell-shape feature (I = 0.4 pA, V_S = 0.2 V). A CO molecule can be observed as a dark depression at the top-left corner, serving here as a height reference in constant-height Δ*f* measurements. **b** and **c**, Constant-height Δ*f* images of this adsorbate (V_S = 0 V, I_{SET} = 0.4 pA, V_{SET} = 0.2 V, and Δ*z* = −2.0 Å and Δ*z* = −1.3 Å for **b** and **c**, respectively). The Δ*f* contrast above CO in **c** shows faint repulsive feature at the center which is similar to the feature at the center of the adsorbate in **b**, if the two images are acquired at a height difference of 0.7 Å. Since in the Pauli-repulsion-imaging regime the contrast is very sensitive to small changes in tip-sample distance, this suggests a height difference between CO and the adsorbate of about larger than 0.7 Å. This is consistent with acetic acid standing upright on the surface. The AFM images also show dumbbell-like features, which might be due to the methyl and hydroxyl groups, while the carbonyl group anchors to the substrate. All scale bars refer to 5 Å.

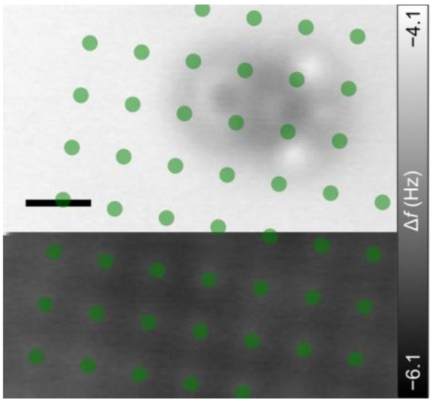

Supplementary Figure 3. Adsorption-site determination of ReX-NHC. The bond-resolved upper part of the image (showing ReX-NHC) was acquired with Δz = +0.46 Å, whereas the lower part of the image, featuring atomic resolution of NaCl, was acquired with Δz = +2.66 Å. Scale bar refers to 5 Å. The Cl atomic sites were identified in the lower part of the image and the resulting grid is indicated by green circles. The center of the central 6-membered ring of ReX-NHC was centered between four green circles, hence, above the sodium sites. This geometry is consistent with the carbonyl groups anchoring to the sodium sites and with the lowest-energy geometry according to the DFT calculations, which is shown in Figure 2e. The constant-height Δf image was measured with the sensor that was also used for Figure 2a.

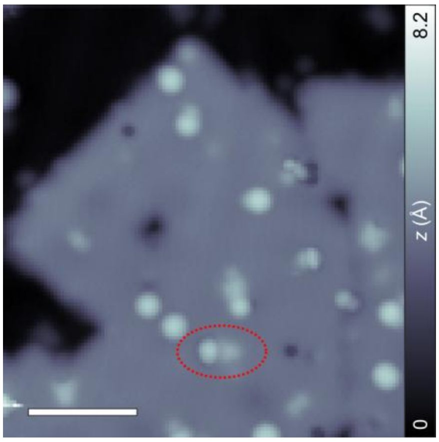

Supplementary Figure 4. Sample overview. Constant-current STM image (I = 1.0 pA, V_S = 0.2 V) acquired after co-depositing Au adatoms. The Au adatoms appeared as circular protrusions with an apparent height about 2.4 Å. After the deposition of Au adatoms, Au-ReX-NHC complexes were observed. An Au-ReX-NHC is marked by a red-dashed oval. These structures were found and characterized seven times. Scale bar refers to 5 nm.

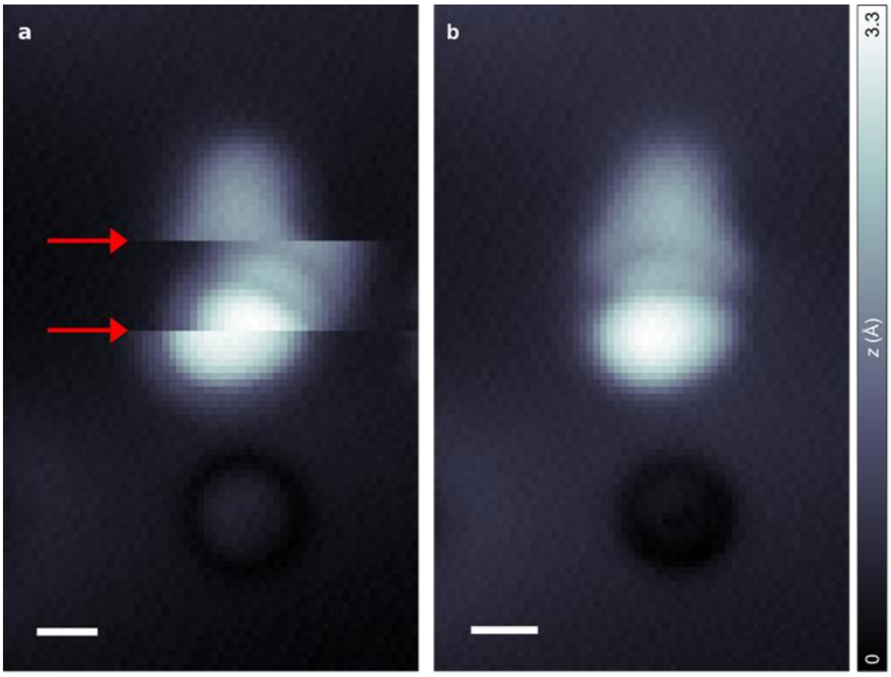

Supplementary Figure 5. STM manipulation of Au-ReX-NHC: Rotation. **a**, Constant-current STM image showing an Au-ReX-NHC that was inelastically excited at the scan parameters of V_S = −2.0 V, I = 0.5 pA. A clear displacement of Au-ReX-NHC is visible from the abrupt contrast change at two scan lines (red arrows). **b**, Constant-current STM image acquired after applying several STM-manipulation experiments. Neither Au charging nor bond breaking was observed (V_S = −1.0 V, I = 0.5 pA).[4,5] A CO-functionalized tip was used in the measurements. All scale bars refer to 5 Å.

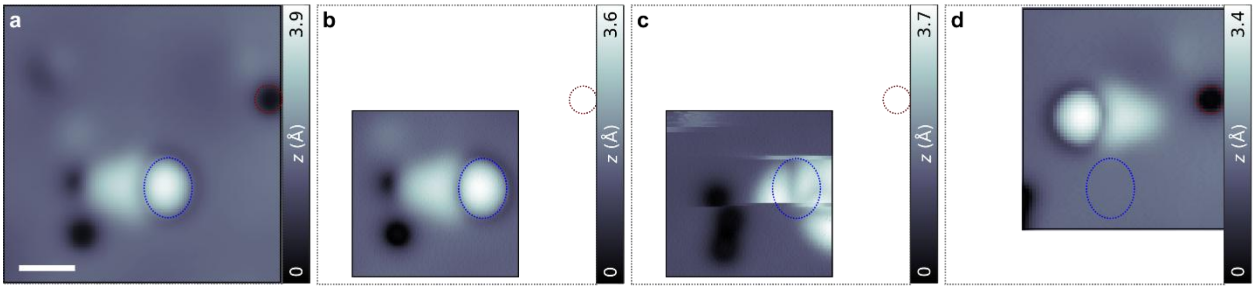

Supplementary Figure 6. STM manipulation of Au-ReX-NHC: Lateral displacement. **a**, Constant-current STM image of an Au-ReX-NHC and CO molecules adsorbed on 2 ML NaCl/Au(111) (I = 0.5 pA, V_S = 0.2 V). The initial position of the Au atom in the Au-ReX-NHC is marked by a dashed-blue ellipse. The CO molecule at the top right serves as a position reference (dashed-red circle). **b**, Constant-current STM image showing the Au-ReX-NHC before manipulation (I = 0.5 pA, V_S = 0.2 V). **c**, Constant-current STM image showing the Au-ReX-NHC during inelastic excitation at the scan parameters of V_S = +1.4 V, I = 0.5 pA. **d**, Constant-current STM image after the manipulation process, showing both a 180° rotation and a lateral displacement of the Au-ReX-NHC with respect to **a** (I = 0.5 pA, V_S = 0.2 V). The Au-ReX-NHC remains intact. Since the field of view changes from **a** to **d,** the one of **a** is indicated by a dashed-grey square in all panels**.** Scale bar refers to 1 nm. A Gaussian filter with standard deviation of 2 was applied to **a**.

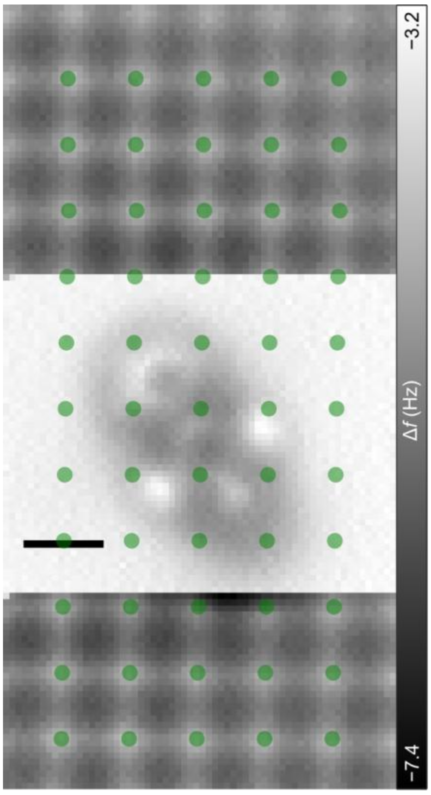

Supplementary Figure 7. Adsorption-site determination of Au-ReX-NHC. The bond-resolved middle part of the image (showing Au-ReX-NHC) was acquired with Δz = −1.2 Å, whereas the upper and lower part of the image, featuring atomic resolution of NaCl, was acquired with Δz = +2.0 Å. Scale bar refers to 5 Å. The Cl atomic sites are indicated by green circles. The result agrees with the DFT calculation, shown in Figure 3f. The constant-height Δf image was measured with the sensor used for Figure 3b

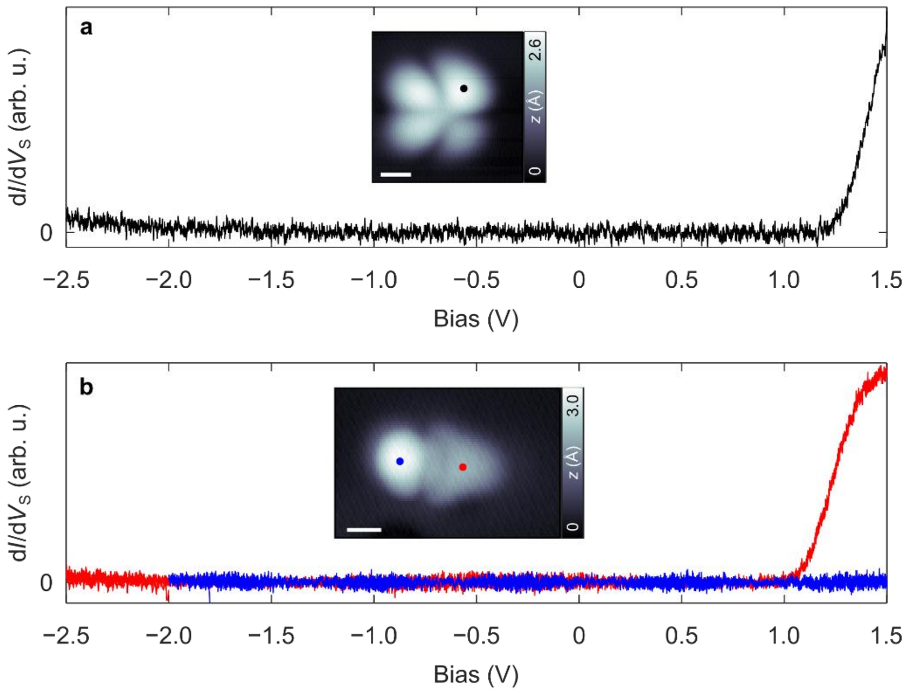

Supplementary Figure 8. Scanning tunneling spectra of ReX-NHC and Au-ReX-NHC. **a**, Differential conductance (dI/dV_S) as a function of sample bias acquired on the ReX-NHC adsorbed on 1 ML NaCl/Au(111). Inset: Constant-current STM image shows the negative ion resonance of ReX-NHC (I = 0.5 pA, V_S = 1.5 V). The feedback loop was opened above the ReX-NHC (black dot in the inset) with parameters of I_{SET} = 5 pA, V_{SET} = 1.5 V. **b**, dI/dV_S spectra acquired above the Au atom and the ReX-NHC moiety of the Au-ReX-NHC complex adsorbed on 2 ML NaCl/Au(111). Inset: Constant-current STM image showing the Au-ReX-NHC (I = 0.5 pA, V_S = −1.0 V). The feedback loop was opened above the ReX-NHC moiety (red dot in the inset) with parameters of I_{SET} = 0.5 pA, V_{SET} = −2.0 V. The corresponding tip positions are indicated by colored dots in the insets. All scale bars refer to 5 Å.

4. Computational Details

All calculations were carried out using the Vienna Ab Initio Simulation Package (VASP),[6–10] employing optPBE-vdW exchange-correlation function.[11] Projector-augmented wave (PAW) pseudopotentials were used,[12–14] and a plane-wave cutoff energy of 600 eV was applied. Electronic states were sampled using Gaussian smearing with a width of 0.1 eV. The NaCl(100)/Au(111) surface was modeled comprising two NaCl atomic layers and one Au layer, with the NaCl layers being relaxed. Convergence criteria were set to 10^{-8} eV for the electronic relaxation and 0.015 eV/Å for the ionic forces during geometry optimization. Molecules are adsorbed on one side of the slab, and dipole corrections to the energy are applied accordingly. A 2×2×1 Monkhorst-Pack k-point mesh was used for Brillouin zone sampling. For the comparison of the calculated orbitals with the measurements at constant-height, the electron density corresponding to the lowest unoccupied molecular orbital (LUMO) or singly-occupied molecular orbitals (SOMO) was integrated along the axis perpendicular to the surface starting from approximately 3 Å above the molecule. The integration was carried out over a distance of 2.4 Å. Increasing the integration range did not show a qualitative change of the results. The integrated density was then mapped and plotted parallel to the surface plane. Charge density maps were visualized using a power-law color normalization to enhance contrast. The integrated charge density was mapped with $\gamma = 1.5$ (PowerNorm) in matplotlib.

4.1 Additional Computational Data

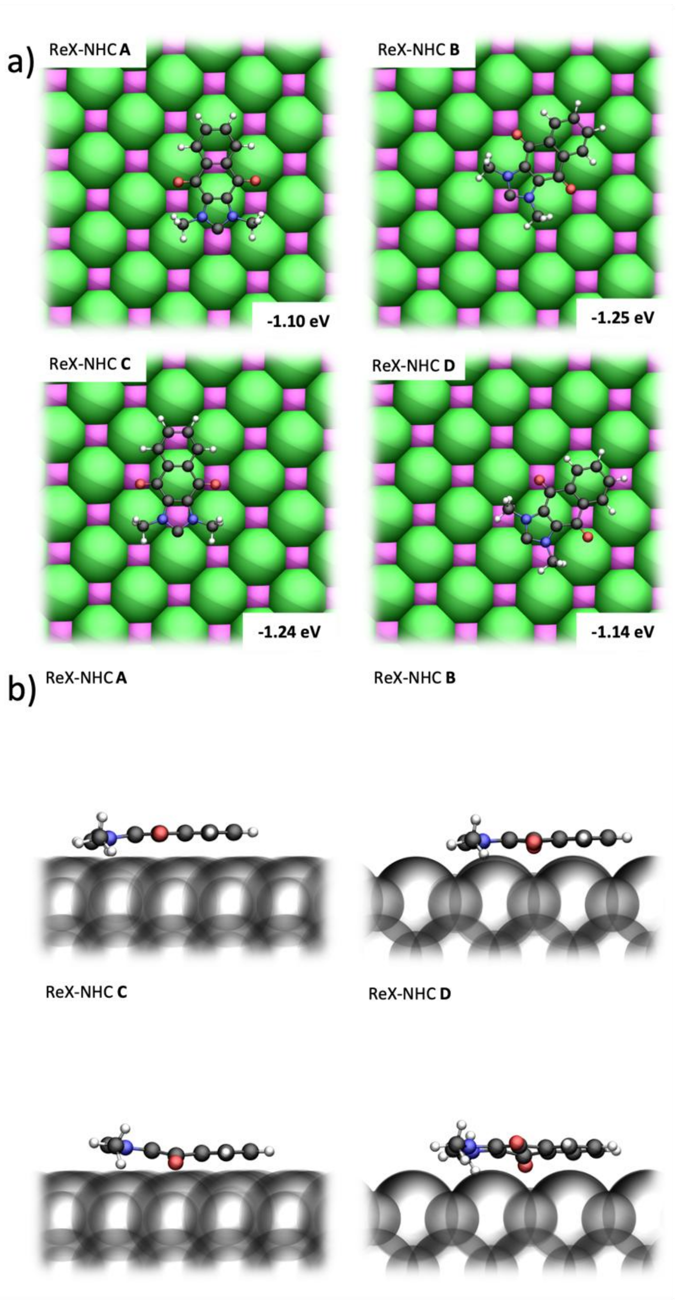

Supplementary Figure 9. Ball-and-stick model of the DFT-optimized adsorption geometries of ReX-NHC on NaCl (100) / Au (111) surface at different adsorption sites, together with the corresponding adsorption energies a) top and b) side view. The adsorption configurations differ with respect to i) the interaction site of the carbene carbon atom with the Na atom (A and B) or with the Cl ion (C and D) and ii) the molecular orientation along the non-polar (a and c) or polar (b and d) direction of the NaCl surface.

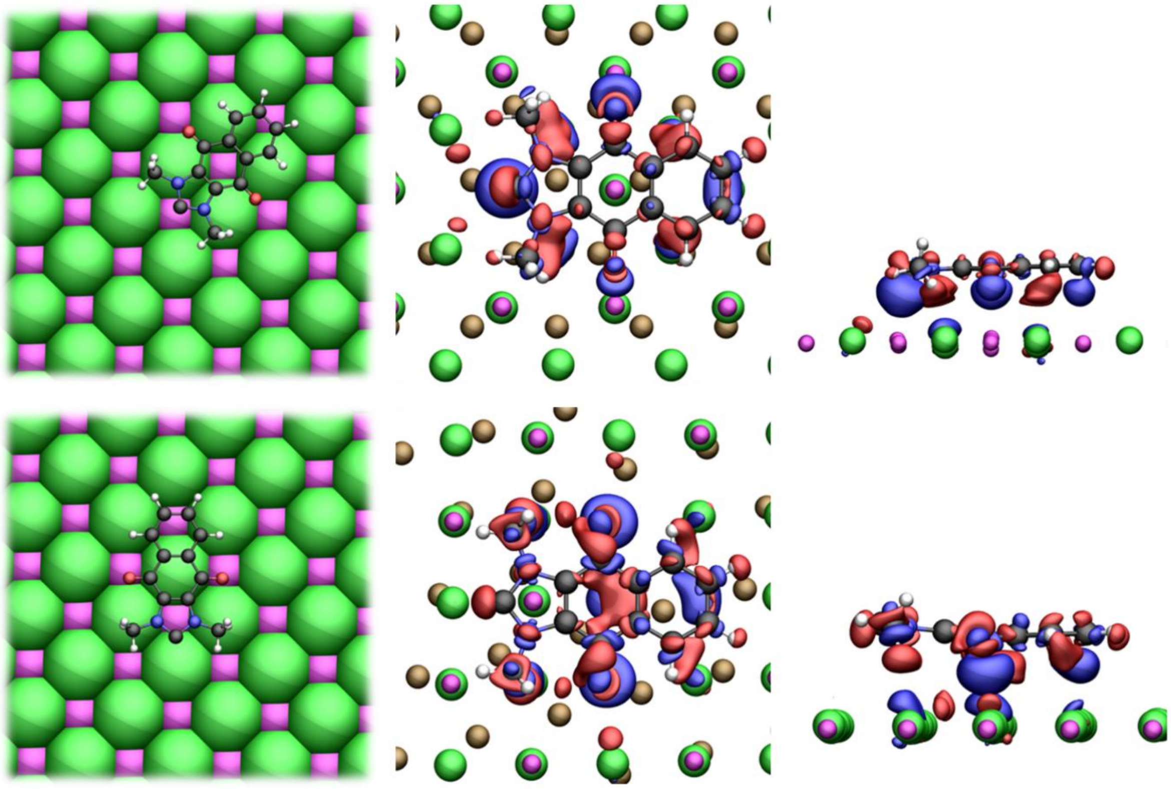

Supplementary Figure 10. Top and side view of the calculated charge redistribution (charge difference) upon adsorption of ReX-NHC on NaCl/Au(111) at two different adsorption sites (blue: increase, red: decrease).

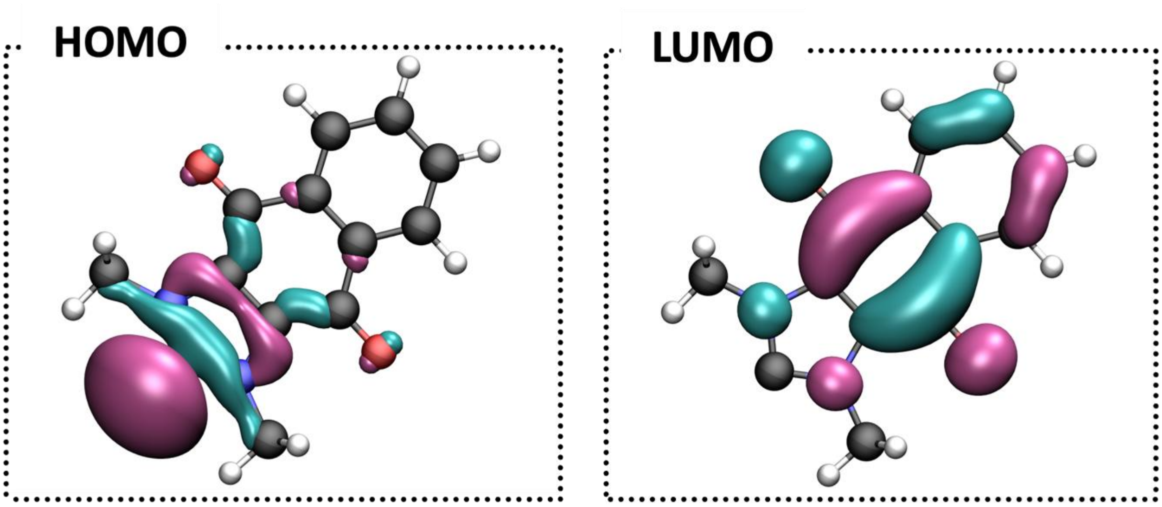

Supplementary Figure 11. Calculated HOMO and LUMO orbitals of the ReX-NHC based on gas-phase calculations.

5. Discussion of Protonation

AFM imaging confirms that the protecting group has been removed. The presence of an additional proton cannot be directly assessed by AFM due to limited sensitivity to single hydrogen atoms. In the low-temperature, ultra-high-vacuum environment of the experiment, no solvent or proton reservoir is present. Adsorption of hydrogen from the residual gas occurs only over long timescales, and its attachment to carbene in all investigated instances seems extremely unlikely.

DFT calculations show that the ReX–NHC acetate precursor dissociates in the gas phase to yield free carbene and acetic acid rather than protonated carbene and acetate, which is in agreement with previously reported calculation on other NHCs.[15]

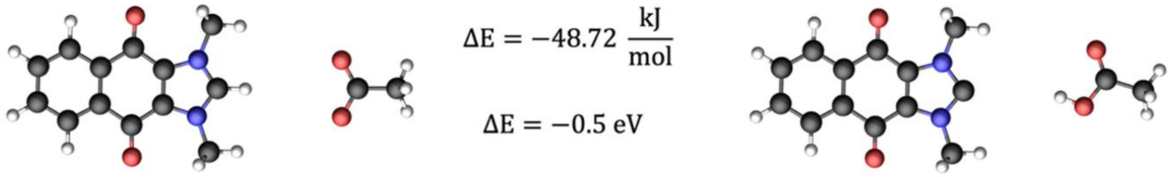

Supplementary Figure 12. Reaction energy for the gas-phase deprotonation of ReX-NHC by acetate.

Taken together with the experimental conditions, these data indicate that the compound exists as the free carbene.

Direct-inlet mass spectrometry of the ReX–NHC acetate precursor (Supplementary Section 1.4) shows a signal at the expected mass for the neutral ReX–NHC upon sublimation, consistent with the above considerations. No dominant signal corresponding to a protonated carbene is observed.

6. Comparison of Simulated and Experimental AFM Images

Density functional theory (DFT) calculations reveal different adsorption sites of ReX-NHC on NaCl (100) / Au (111) with only small energy differences (see Supplementary Figure 9). Importantly for the comparison of the experimental and simulated AFM images, the molecule exhibits configuration-dependent buckling, with all low-lying adsorption geometries differing primarily in the extent of distortion of the otherwise planar molecular backbone. In particular, the carbene carbon can bend either toward or away from the surface, depending on the specific adsorption geometry. (cf. configuration A and C in Supplementary Figure 9). The small energy differences between these configurations indicate a notable degree of flexibility of the adsorbed molecule. We therefore attribute the remaining discrepancies between simulated and experimental AFM images to the inherent limitations of static calculations in fully capturing the experimental conditions. Owing to the high sensitivity of AFM, even moderate geometric distortions significantly affect the simulated contrast, as shown in Supplementary Figure 13. In addition, the relatively shallow potential energy landscape arises from the predominantly noncovalent nature of the molecule–surface interaction, reflecting a delicate interplay between the local attraction of polar functional groups and the energetic penalty associated with molecular buckling. However, even at the density functional theory (DFT) level, including dispersion corrections, these subtle energetic contributions and the associated structural flexibility may not be fully captured, thereby further limiting quantitative agreement between experiment and simulation.

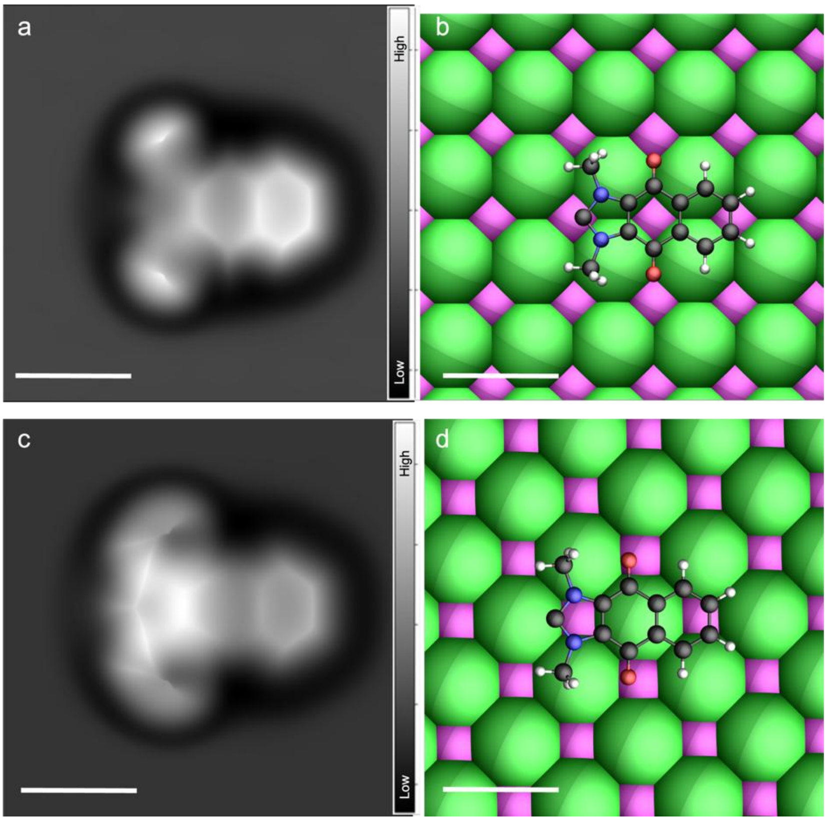

Supplementary Figure 13. Comparison of the simulated AFM images (a,c) for two energetically close-lying adsorption sites of ReX-NHC on NaCl/Au(111) together with the ball-and-stick model of the corresponding optimized geometry (b,d).

7. References

[1] C. Gutheil, G. Roß, S. Amirjalayer, B. Mo, A. H. Schäfer, N. L. Doltsinis, B. Braunschweig, F. Glorius, *ACS Nano* **2024**, *18*, 3043.

[2] F. J. Giessibl, *Appl. Phys. Lett.* **1998**, *73*, 3956.

[3] L. Gross, F. Mohn, N. Moll, P. Liljeroth, G. Meyer, *Science* **2009**, *325*, 1110.

[4] J. Repp, G. Meyer, F. E. Olsson, M. Persson, *Science* **2004**, *305*, 493.

[5] L. Gross, F. Mohn, P. Liljeroth, J. Repp, F. J. Giessibl, G. Meyer, *Science* **2009**, *324*, 1428.

[6] J. P. Perdew, K. Burke, M. Ernzerhof, *Phys. Rev. Lett.* **1996**, *77*, 3865.

[7] G. Kresse, J. Hafner, *Phys. Rev. B* **1993**, *47*, 558.

[8] G. Kresse, J. Hafner, *Phys. Rev. B* **1994**, *49*, 14251.

[9] G. Kresse, J. Furthmüller, *Phys. Rev. B* **1996**, *54*, 11169.

[10] G. Kresse, J. Furthmüller, *Comput. Mater. Sci.* **1996**, *6*, 15.

[11] J. Klimeš, D. R. Bowler, A. Michaelides, *J. Phys.: Condens. Matter* **2010**, *22*, 22201.

[12] G. Kresse, J. Hafner, *J. Phys.: Condens. Matter* **1994**, *6*, 8245.

[13] P. E. Blöchl, *Phys. Rev. B* **1994**, *50*, 17953.

[14] G. Kresse, D. Joubert, *Phys. Rev. B* **1999**, *59*, 1758.

[15] H. Du, X. Qian, *Carbohydr. Res.* **2011**, *346*, 1985.
